# Early integration of palliative care in outpatient nephrology: exploring healthcare professionals’ knowledge, attitudes, and communication practices – an exploratory multi-methods study

**DOI:** 10.1186/s12882-026-05409-8

**Published:** 2026-10-09

**Authors:** Melanie Joshi, Mareike Löbberding, Jana Grimm, Axel Doll, Steffen T. Simon, Raymond Voltz, Linus Völker, Julia Strupp

**Affiliations:** 1 Department of Palliative Medicine, Faculty of Medicine and University Hospital Cologne, University of Cologne, Cologne, Germany; 2 Department II of Internal Medicine, Faculty of Medicine and University Hospital Cologne, University of Cologne, Cologne, Germany; 3 Center for Molecular Medicine Cologne (CMMC), Faculty of Medicine and University Hospital Cologne, University of Cologne, Cologne, Germany; 4 Center for Integrated Oncology Aachen Bonn Cologne Düsseldorf (CIO ABCD), Faculty of Medicine and University Hospital Cologne, University of Cologne, Cologne, Germany; 5 Center for Health Services Research Cologne (ZVFK), Faculty of Medicine and University Hospital Cologne, University of Cologne, Cologne, Germany

**Keywords:** Palliative care, Nephrology, Outpatient care, Advanced kidney disease, End-stage renal disease, Communication skills, Professional education, Implementation science

## Abstract

**Background:**

Patients with advanced kidney disease often experience high symptom burden and complex illness trajectories, highlighting the importance of integrating palliative care into nephrology. However, palliative care remains insufficiently integrated into routine care within nephrology outpatient centers. This study explored healthcare professionals’ (HCPs) perspectives on palliative care integration, including knowledge and confidence in identifying patients with palliative care needs as well as perceived barriers and facilitators to implementation.

**Methods:**

An explorative multi-methods study was conducted as part of a project aiming to implement a minimally invasive intervention in nephrology outpatient centers in Germany (MINI-Neph). HCPs from participating centers received a one-hour training introducing palliative care in nephrology, communication strategies for emotionally challenging situations, and using two validated tools for identifying patients with palliative care needs: the Surprise Question (SQ) and the Supportive and Palliative Care Indicators Tool (SPICT-DE). Data were collected through an online survey, free-text responses and semi-structured interviews with HCPs and analyzed using descriptive statistics and qualitative content analysis, respectively.

**Results:**

Five nephrology outpatient centers located in different regions across Germany were involved in the MINI-Neph-Study. A total of 52 HCPs participated; most were female (82.7%), with the largest age groups being 31–40 years (30.8%) and 51–60 years (28.8%). Roles included nurses (57.7%), medical assistants (25.0%), and nephrologists (15.4%). While most participants recognized the relevance of integrating palliative care into nephrology, only about half reported confidence in identifying patients with palliative care needs in their presumed last year of life. Communication about patients’ preferences regarding care, treatment decisions, and advance directives occurred inconsistently, while discussions about preferred place of care or death were reported less frequently. Knowledge of legal aspects of end-of-life care and confidence in organizing referrals to palliative care services were limited. Additionally, 13 qualitative interviews were conducted with HCPs from three nephrology outpatient centers (2 nephrologists, 2 medical assistants, and 8 nurses). The interviews highlighted the complex and dynamic trajectories of patients with advanced kidney disease. Conversations about serious illness and care goals were mostly reactive, while interprofessional communication and collaboration with external providers remained limited. Training increased awareness of palliative care needs, but structural barriers such as time pressure and a treatment-focused culture hindered consistent implementation.

**Conclusions:**

Early integration of palliative care in outpatient nephrology is valued by HCPs, while confidence in identifying palliative care needs and communicating about end-of-life issues was inconsistent, and palliative care remains predominantly reactive in routine care. The MINI approach may support more systematic and proactive care. More proactive care may require protected time, structured communication, clear responsibilities, and established communication pathways.

**Supplementary Information:**

The online version contains supplementary material available at https://doi.org/10.1186/s12882-026-05409-8.

## Introduction and background

Due to demographic changes and improved life expectancy, chronic kidney disease (CKD) is a growing global health problem in a multimorbid and frail patient cohort [1, 2]. In Germany, nearly 13% of adults suffer from CKD, with prevalence rising sharply in older age groups; one in eight adults aged 70–79 years is affected, and prevalence is likely higher in those over 80 years [3]. Older patients with advanced CKD represent the fastest-growing dialysis population and face markedly reduced life expectancy, multiple comorbidities, and a high symptom burden [4, 5]. End-stage renal disease (ESRD) dramatically shortens survival, with approximately 50% of patients dying within five years of initiating renal replacement therapy [6]. Acute kidney injury (AKI) is both a risk factor for and a complication of CKD, with around 10% of AKI patients remaining dialysis-dependent, and both conditions increase the risk of cardiovascular and other comorbidities [7, 8].

Patients with advanced kidney disease experience a high symptom burden due to the disease or treatment, including fatigue, pruritus, nausea, pain, and psychological distress [9]. Dialysis, while life-sustaining, often contributes to decreased quality of life [10]. Despite this, palliative care is frequently introduced late or not at all [11–13], and dialysis is initiated even in cases with markedly reduced prognosis due to old age, multimorbidity, frailty, and lack of access to kidney transplantation. In addition to the renal-specific Bansal and REIN scores supporting decision-making in patients over 80 years and progressive CKD, tools established in palliative care can help identify high-risk patients [2].

Early palliative care aims to alleviate physical and psychological symptoms, improve quality of life, and support patients and families through shared decision-making [14, 15]. For patients with AKI, CKD, and ESRD, integrating palliative care ideally occurs alongside nephrological treatment, before complications arise, and continues throughout the disease trajectory [16, 17]. However, there is limited consensus on the timing and approach to palliative care in non-malignant kidney disease, and awareness among patients and healthcare providers varies [16, 18].

In Germany, hemodialysis in nephrology outpatient centers (nephrology practices with affiliated dialysis centers) continues to be the predominant form of treatment [19]. Often, they are independent clinics that are not affiliated with a hospital and specialize in providing outpatient dialysis.

Our study aimed to explore HCPs’ perspectives on palliative care integration in outpatient nephrology, assess knowledge and confidence in identifying and supporting seriously ill patients, and to identify barriers and facilitators to implementing structured palliative care approaches.

## Methods

### Study design and context

**Fig. 1:**
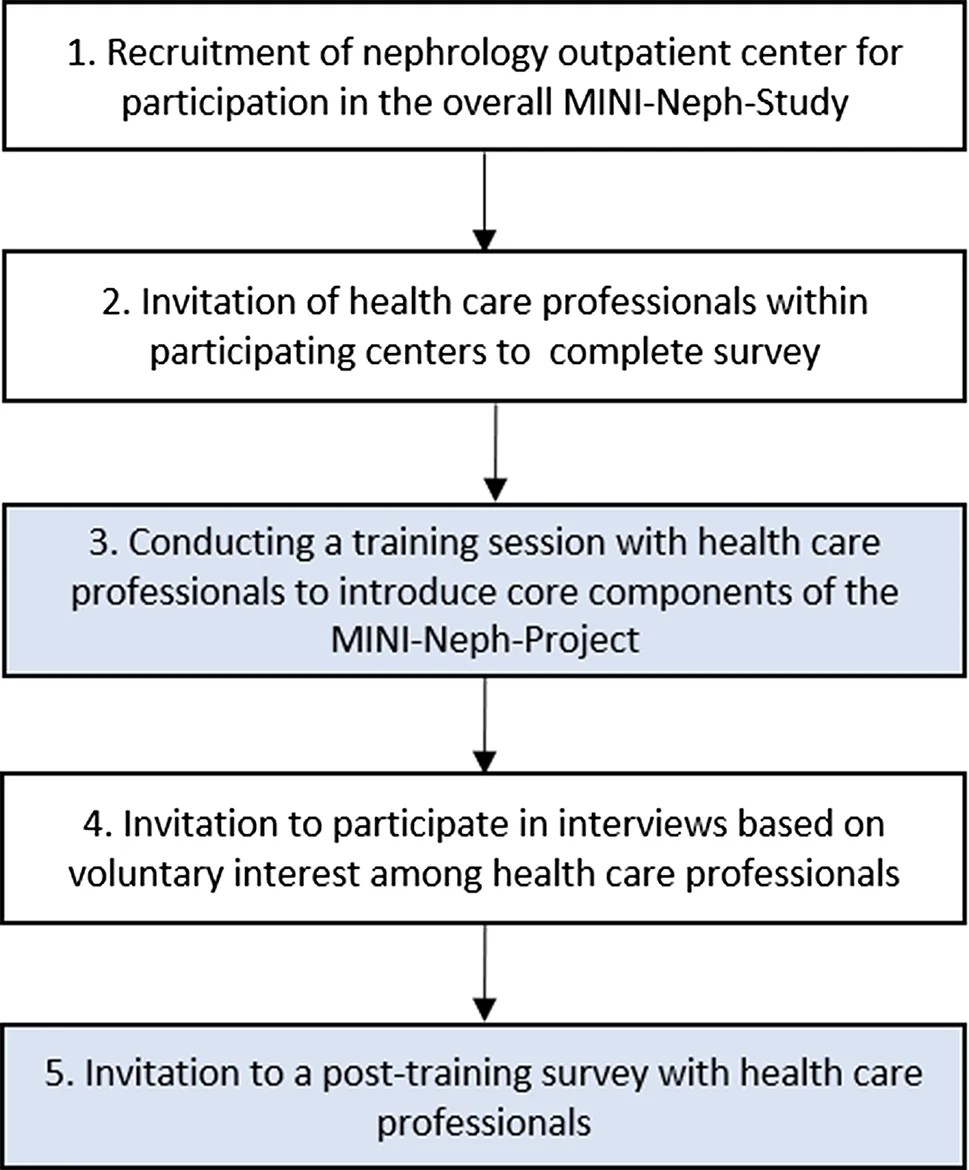
Study design of the overall study

This article describes the cross-sectional online survey followed by qualitative semi-structured interviews as part of a project aiming to implement a minimally invasive intervention (MINI) in nephrology outpatient centers. Our multi-methods approach aimed to achieve greater validity of the results by combining and discussing consistency of the data across different approaches [20], see Fig. 1 for the overall study design. Quantitative data was used to assess self-reported competencies and processes in current practice whereas qualitative data were used for personal experiences, perspectives on high-quality care for terminally ill patients in nephrology, and expectations regarding the integration of palliative care in general. This is reported in consolidation with the STROBE [21] and COREQ [22] guideline.

### Recruitment, inclusion and exclusion criteria

Nephrology outpatient centers were recruited using a pragmatic convenience sampling approach. We approached existing professional networks across Germany, with the support of the Commission for Palliative Medicine of the German Society of Nephrology (Deutsche Gesellschaft für Nephrologie – DGfN). We selected centers from different regions (North Rhine-Westphalia, Saxony, Lower Saxony) in Germany to capture a broader range of perspectives. Recruitment was conducted from November 2023 to October 2025 via email and telephone, supplemented by informational materials describing the study’s aims and procedures and an informational kick-off meeting.

After nephrology outpatient centers agreed to participate, each received a welcome package prior to the training. The package included study information, a printed poster displaying the QR code for the pre-training online survey, and non-monetary incentive (coffee and chocolate). Participation was voluntary, so, to encourage pre-training participation, a regular reminder with the QR code was sent out, and the option was given to complete the online survey 15 min before the start of the training. Thus, the survey was administered prior to the intervention and was not influenced by the training. After completing the online survey, the participants were given the option to provide their contact details for a qualitative interview. The interviews were conducted after the training and the implementation of the MINI in the centers and captured participants’ experiences and perceptions following the intervention. The researchers first established contact with the participants within the context of the study via telephone and email. A target of 10–20 interviews was defined based on the literature on data saturation [23].

Inclusion criteria were being employed in a participating nephrology outpatient center with direct patient care responsibilities, being of legal age, providing informed consent and having sufficient German language skills to complete the questionnaire. Participants who did not meet any of these criteria were excluded from the study.

### Training and intervention

Participating nephrology outpatient centers received a one-hour training session delivered by a researcher and a HCP in Palliative Care and Nephrology, respectively, either on-site or virtually via Zoom. Training content included the role of palliative care in nephrology, palliative and hospice care structures and information on supporting networks, and communication strategies in emotionally challenging situations (SPIKES protocol [24] and NURSE model [25]). Written materials, such as pocket cards, were also provided to support the implementation of the training content in clinical practice. As part of the training, participants were introduced to the minimally invasive intervention (MINI), designed to identify patients with palliative care needs in the presumed last year of life based on two validated tools: the Surprise Question (SQ) (“Would I be surprised if this patient were to die in the next 12 months?”) and the Supportive and Palliative Care Indicators Tool (SPICT) [26–28]. While the SPICT has shown slightly higher sensitivity than the SQ in identifying patients with unmet palliative needs (57% vs. 50%) [29, 30], both tools are valuable in supporting clinical judgment, particularly among non-specialist healthcare professionals (HCPs) [31]. Further information on the minimally invasive intervention is available in previous publications [28, 27].

**Fig. 2:**
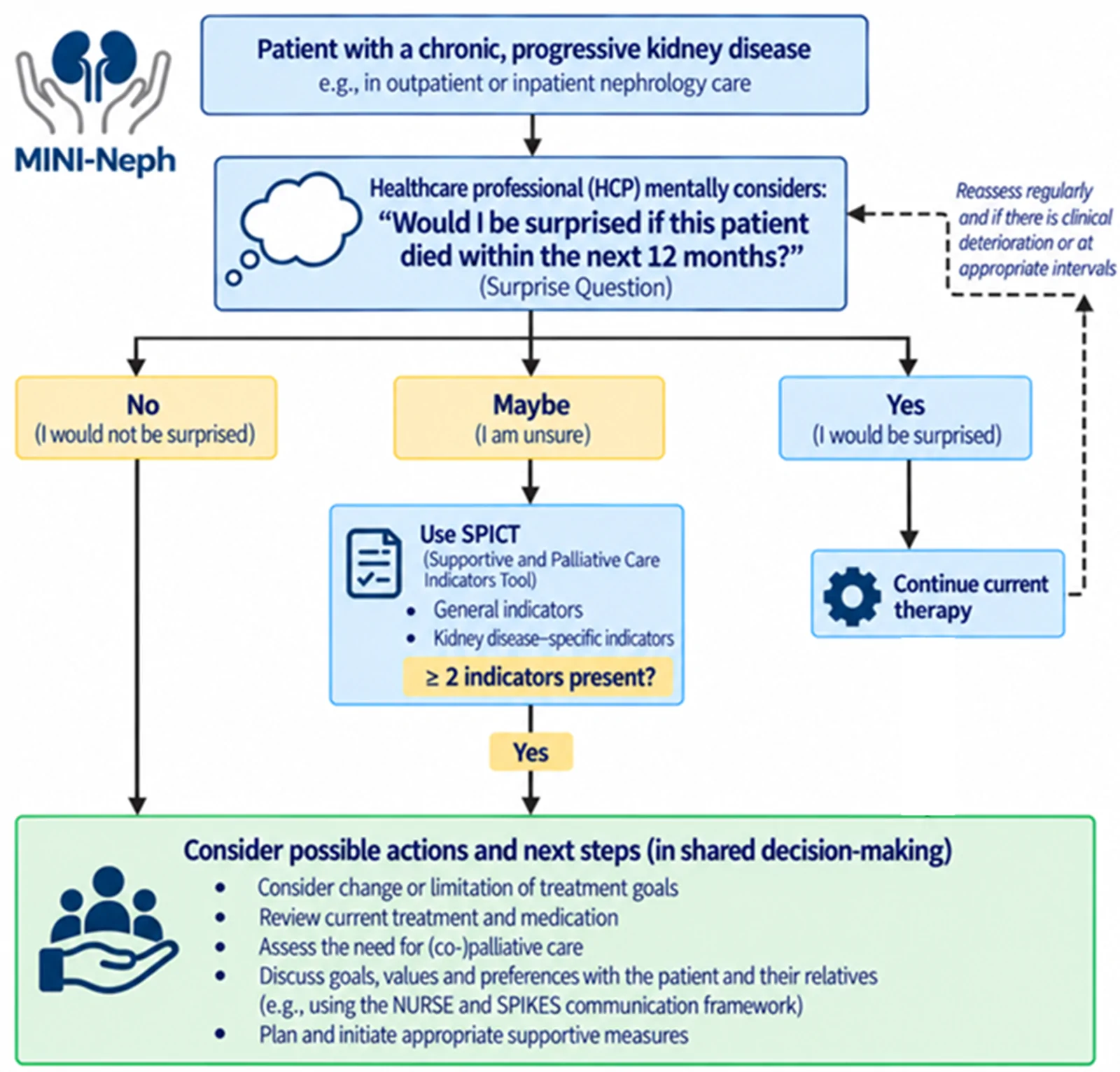
Overview of the MINI and its components

Following the training, participants were asked to apply the MINI components in their clinical practice, including the SQ and SPICT, and to use the communication strategies introduced during the training when discussing palliative care needs with patients. Figure 2 provides an overview of the MINI and its components.

### Data collection, instruments and analysis

#### Online survey: quantitative data and free-texts

HCPs from participating nephrology outpatient centers were surveyed using a self-developed questionnaire, available online via QR-Code or direct link from April 2024 until June 2025. The online survey was implemented via the *LimeSurvey* platform hosted at the University of Cologne (https://www.limesurvey.org/de).

The questionnaire was based on the German National S3 Guideline for Palliative Care [32] and previous findings from the LYOL-C I and II studies [27, 28]. It collected sociodemographic data and self-assessments across three dimensions: (1) self-reported palliative care confidence including the ability to identify patients likely to be in their last year of life, (2) assessment of the current situation regarding communication about patients’ preferences and needs and responsibility for end-of-life discussions, and (3) the initiation and role of palliative care in patients with advanced kidney disease. Palliative care confidence was assessed based on 10 of the 18 items of the Palliative Competence Test (PKT), originally validated for physicians in an adapted version due to subject relevance [33]. Items concerning communication about dying and death were based on the confidence questionnaire used in a previous study [34], with wording and response formats adapted to allign with the rest of the questionnaire.

Responses regarding confidence were recorded on a 4-point Likert scale, with an additional “not applicable” or “no answer” option, respectively. For the assessment of issues related to dealing with the desire to die, a 5-point Likert scale was used [35]. Confidence related to communication about the desire to die with patients was self-assessed on a 7-point scale (1 least confidence to 7 highest confidence). We also used open-ended free-text questions (“In your opinion, what are the advantages of palliative care for patients with incurable advanced kidney disease?”, “Palliative care has so far played a rather minor role in the treatment of patients with incurable advanced kidney disease. What do you think are the possible reasons for this?“ and “Is there anything else you would like to share with us regarding the care of patients with serious/incurable illnesses?”). The overall questionnaire was not validated in our study; however, several items had been validated in another study [33] and questionnaire comprehension and usability were assessed for face validaty through a *think-aloud* pretest with staff from the Department of Palliative Medicine, University Hospital of Cologne. Following feedback, minor adjustments to wording and format were made.

The quantitative data were analyzed descriptively using IBM SPSS Statistics 30. For a solely exploratory and descriptive analysis, the responses to the Likert scales were analyzed in a dichotomous format (“Strongly disagree” and “disagree” vs. “strongly agree” and “agree” (and “partially agree”, when applicable)). The response options “not applicable” or “no answer” were treated as valid values, whereas missing values were not reported, resulting in varying denominators across outcomes (item-level missingness).

Free-text responses were analyzed using Microsoft Excel 2024. Given the brevity of the responses and the nature of the free-text format, the participants’ statements were coded inductively using content analysis [36]. Ten free-text responses were used to establish categories for an initial codebook. Each text segment containing relevant content was assigned to a category inductively. Every further text segment was evaluated to determine whether it could be subsumed under an existing category. Only when no suitable category existed was a new category established, thereby maintaining exhaustivity. To maintain exclusivity, no multiple coding was allowed. After coding, the frequency of each category was counted to assess its overall relevance.

#### Qualitative interviews

A convenience subsample of participants was recruited, aiming to include professionals from different nephrology outpatient centers and occupational groups (physicians, nurses, medical assistants) from March to August 2025. The interviews explored participants’ perspectives on high-quality care for terminally ill patients in nephrology (e.g. reflections on communication with patients and families), experiences with the integration of palliative care in general (e.g. perceived facilitators and barriers), and experiences with MINI implementation. An interview guide was developed by the research team based on the objectives of the study (see Supplementary Material 1). Prior to the interview, participants were asked to provide sociodemographic and professional characteristics. The qualitative interviews were subsequently conducted via telephone by MJ and ML, both female, registered nurses and health services researchers experienced in qualitative methodology. All interviews were audio-recorded and transcribed verbatim. Field notes were documented during and after the interviews to capture relevant contextual information. Interview data were analyzed using structuring content analysis [37]. The analysis followed a systematic process comprising familiarization with the interview transcripts, application and refinement of deductive main categories, development of inductive subcategories, and independent coding with subsequent consensus discussions. For data management and analysis MAXQDA-Software (VERBI Software, versions 2024 and 2026) was used. ML and MJ conducted the interviews and were subsequently involved in the analysis. Prior to coding, both researchers independently familiarized themselves with the interview transcripts through repeated reading to gain an overview of the data and identify relevant content. Deductive main categories were derived from the interview guide, with inductive subcategories developed from the data. Three interviews from different centers and occupational groups were independently coded deductively by the two first authors (ML, MJ) to test and refine the category system. The coding was subsequently discussed until consensus concerning coding rules and categories was reached. All interviews were independently coded by either ML or MJ and re-coded by the other researcher, respectively. Coding discrepancies were resolved through discussion until consensus was reached. This iterative process of independent coding, comparison, and consensus discussion was used to enhance the credibility and dependability of the analysis and to reduce the influence of individual interpretations. The coding framework is described in the supplementary materials. Within the content analysis, barriers and facilitators related to MINI implementation and the integration of palliative care were identified. After completion of the content analysis, these identified barriers and facilitators were mapped to the corresponding Consolidated Framework for Implementation Research (CFIR) domains to contextualize the findings within an implementation science framework. This mapping included both aspects related to the implementation of MINI and broader factors relevant to the integration of palliative and end-of-life care.

All quotes were translated into English using DeepL Translate (Version 2026). The translations were back-translated into German and checked against the original quotes by a second team member to ensure accuracy and preservation of the original meaning. Where necessary, translations were slightly adjusted for clarity while preserving the original meaning.

### Ethical considerations

The study was conducted in accordance with the Declaration of Helsinki and approved by the Ethics Committee of the Medical Faculty at the University of Cologne (#23-1246). The trial was registered with the German Clinical Trials Register (#DRKS00033063 (22.12.2023)). Participation was voluntary. Participants received written information about the study. They were informed about the researchers’ roles and the purpose of the study. Consent was indicated via a checkbox for the online survey, and written informed consent was obtained for the semi-structured interviews prior to enrolment. In the online survey, participants generated a personal code known only to themselves to allow for the optional withdrawal of their data. Interview transcripts were pseudonymized prior to analysis to protect sensitive information. They were not returned to participants for comment or correction. Transcripts were coded using a number for the respective nephrology outpatient center and a consecutive number for each interview.

## Results

### Online-survey

#### Participant characteristics

A total of 52 HCPs from five nephrology outpatient centers participated in the survey. Participants represented a range of professional backgrounds, including nephrologists, nurses, and medical and nursing assistants. The majority were female (82.7%). Participants had varying levels of experience in nephrology care, with the largest group reporting 11–20 years of experience (see Table 1).

**Table 1:** Sample characteristics (online survey)

Characteristic			
	Total (*n* = 52)		
		*n*	%
Age	< 20 years	1	1.9
	21–30 years	6	11.5
	31–40 years	16	30.8
	41–50 years	9	17.3
	51–60 years	15	28.8
	> 60 years	5	9.6
Gender	Male	9	17.3
	Female	43	82.7
	Diverse / Other	0	0
Professional group	Nephrologist	8	15.4
	Nursing HCP	30	57.7
	Medical assistant Nursing assistant	13 1	25 1.9
Experience in nephrology care	< 1 year 1–5 years	7 9	13.5 17.3
	6–10 years	7	13.5
	11–20 years > 20 years	17 12	32.7 23.1
Experience in caring for patients with serious/incurable illness	< 1 year 1–5 years 6–10 years 11–20 years > 20 years	5 8 6 17 16	9.6 15.4 11.5 32.7 30.8

#### Confidence and identification in caring for patients in their presumed last year of life

A total of 23 participants (57.5%) (strongly) agreed that they were familiar with criteria indicating that patients are likely in their last year of life, while 17 (42.5%) (strongly) disagreed. Regarding confidence in identifying patients who may be in their last year of life, 20 participants (51.3%) reported feeling confident, whereas 19 (48.7%) (strongly) disagreed. Confidence in caring for patients who were presumably in their last year of life was agreed (strongly) by 23 participants (59.0%) while 16 participants (41.0%) (strongly) disagreed.

#### Current practice, responsibility, and perceived confidence and ability in end-of-life communication

Regular communication with patients who were presumably in their last year of life regarding their preferences and needs in different aspects of care as well as communication within and across teams, was reported to vary among participants (see Table 2).

**Table 2:** Patient and team communication practice

Item / Question	Responses (*n*)	Strongly agree/ agree (%)	Strongly disagree /disagree (%)	Not applicable/Not specified (%)
**Patient and relative communication**				
I feel confident to communicate to patients and their relatives that **the illness is incurable and life-threatening**	39	46.2	46.2	7.7
*I regularly discuss their preferences and needs with patients who are likely in the last year of their lives regarding…*				
…their desired type of care	39	48.7	38.5	12.8
…their preferred place of care and death	38	26.3	63.2	10.5
…upcoming treatment decisions	38	44.7	47.4	7.9
…an advance directive	38	52.6	34.2	13.2
…a health care proxy/power of attorney	38	47.4	39.5	13.2
There is regular communication regarding their preferences and needs with patients’ relatives	38	50.0	31.6	18.4
**Interprofessional communication**				
*There is regular communication regarding preferences and needs of patients who are likely in the last year of their lives…*				
…within the team (e.g., in team meetings)	39	33.3	66.7	---
…with treating specialists (e.g., cardiologists, general practitioner (GP))	38	23.7	52.6	23.7
…with specialized palliative home care teams	38	21.1	60.5	18.4

Participants rated their perceived ability to address specific patient needs and ability in challenging conversations (see Table 3).

**Table 3:** Self-perceived ability to address patient needs and manage challenging conversations

Item / Question	Responses (*n*)	(Strongly) agree (%)	(Strongly) disagree (%)	Not specified (%)
**Ability to address patient needs**				
*I think that I am capable of…*				
…collecting data that describe a patient’s pain in a differentiated way	36	55.6	33.3	11.1
…presenting the regional offers of specialized palliative care	36	27.8	63.9	8.3
…convincing a colleague of the need for palliative support	36	58.3	36.1	5.6
…recognizing psychosocial problems and discuss them with patients and relatives	36	47.2	44.4	8.3
…organizing a referral to hospice and palliative care services	36	44.4	50.0	5.6
…recognizing the complex needs of a dying person and to react appropriately to them	36	41.7	50.0	8.3
**Ability in challenging conversations**				
*I think that I am capable of…*				
…remaining in conversation when the wish for euthanasia is expressed^1^	36	55.6	33.3	11.1
…integrating cultural and spiritual aspects of dying and death into the treatment of seriously ill people	36	33.3	50.0	16.7
…recognizing whether a person is suffering, even if the possibilities of communication are limited	36	72.7	25.0	2.8
…advising a patient on the framework of the living will and to draw one up with him/her	36	27.8	63.9	8.3

^1^Due to the study’s location in Germany and use of a German questionnaire, this question is based on the applicable legal framework, which does not allow euthanasia. Here, “remaining in conversation” refers to continuing to engage with patients when they express a wish for euthanasia, taking this wish seriously and addressing it openly rather than avoiding or ending the conversation

It was considered difficult for patients to express fears and concerns about dying and death during communication with physicians by 30 participants (76.9%), whereas six participants (15.4%) (strongly) disagreed with this statement.

Palliative care services were most frequently considered responsible for conducting conversations about dying and death with patients and their relatives by 13 participants (33.3%), followed by GPs, selected by 21 participants (28.2%), and the treating specialist in outpatient care by nine participants (23.1%). Hospital physicians were referred to by three participants (7.7%), while nurses, psychologists, others and “everyone” were selected by one participant (2.6%) respectively.

A total of 35 participants reported moderate confidence in discussing death-related issues with patients (median = 4 on a 7-point scale), whereas 13 participants (37.1%) indicated agreement that time limitations prevented them from discussing a desire to die. Regarding knowledge of the current legal framework regarding assisted suicide/assisted dying, withholding or withdrawing treatment, 35 respondents (74.3%) (strongly) disagreed to the statement “I know the current legal situation”, and only nine participants (25.7%) reported partial or full knowledge.

#### Initiation and role of palliative care in patients with incurable nephrological diseases

A total of 21 participants (58.4%) (strongly) agreed that palliative care should be initiated at the time of diagnosis of an incurable nephrological disease, while 8 participants (22.2%) (strongly) disagreed. Regarding the perceived benefits of early palliative integration, 23 participants (44.2%) provided free-text responses. Reported benefits comprised addressing patient needs including symptom management and psychological support, enabling conversations about death and dying and distributing workload emotionally. Reasons why palliative care currently plays a minor role in their field, were explained by 22 participants (42.3%) who identified a number of factors, including lack of awareness of palliative care possibilities, focus on nephrological diagnosis and dialysis, insufficient communication with specialists, time constraints, and patient protection.

### Qualitative interviews

A total of 13 HCPs participated in the interviews. The demographic and occupational data of the interviewees are listed in Table 4. Mean interview duration was 17.8 min (11.25–30.48 min).

**Table 4:** Sample characteristics (interviews)

Characteristic				
		*n*	%	Mean (Range)
Age (years); *n* = 12				45.5 (31–61)
Gender; *n* = 13	Male	2	15	
	Female	11	85	
	Diverse / Other	0	0	
Professional group; *n* = 13	Nephrologist	2	15.4	
	Nurses	9	69.2	
	Medical assistant	2	15.4	
Experience in nephrology care (years); *n* = 12				19.4 (10–30)
Palliative care training (not MINI-Neph); *n* = 12	Yes No	3 9	25 75	
Access to intervention materials; *n* = 12	Yes No	8 4	66 33.3	

Six categories emerged from the interview-data: “complex and dynamic patients’ conditions”, “conversations about serious illness and palliative needs”, “therapy goal adjustments”, “team communication”, “collaboration with external actors”, and “MINI implementation”. The coding framework is provided in the Supplementary Material 2. Barriers and facilitators for the MINI implementation and the integration of palliative care are structured according to the CFIR domains (see Table 5), capturing both MINI-specific and broader contextual aspects relevant to palliative and end-of-life care. The corresponding CFIR constructs and supporting citations are provided in the Supplementary Marterial 3.

#### Complex and dynamic patients’ conditions

HCPs described patients in nephrology outpatient centers as a heterogeneous group in terms of age, disease progression, and comorbidities. They reported that an increasing number of older patients and patients with oncological diseases are being treated. Many patients show a progressive deterioration in their general condition and indicate that the patients’ condition is characterized by dynamic changes. The state of health may deteriorate during certain phases, while stabilizing or improving during others.

*This is the case with dialysis patients*,* who have been with us for years and whose serious illness also varies in severity*,* meaning that it can worsen or improve during a certain phase*,* or the patient may also develop secondary conditions that worsen or improve their condition. (Physician*,* P02_01*,* Pos. 9)*

#### Conversations about serious illness and palliative needs

HCPs notice that physical strain is often discussed by patients during consultations. This includes pain during transport or dialysis, loss of appetite, lack of energy at home, and side effects of medication and dialysis:

*So basically*,* pain is an issue. Pain during transport*,* pain from lying down for long times during dialysis*,* loss of appetite*,* no energy at home […] (Physician*,* P01_06*,* Pos. 5)*

Within these conversations, existential concerns may also emerge, particularly when the progression of the illness or the end-of-life is discussed. Patients may ask how long they can expect to live after stopping dialysis and what the dying process may involve:

*Until the patients pass away. Between attempting to discontinue dialysis and passing away*,* because many want to know that too […] (Physician*,* P01_06*,* Pos. 15)*

Questions and uncertainties regarding dialysis treatment can also become part of these conversations. For example, patients may ask how long dialysis will be necessary or whether the treatment can be stopped:

*And then he said to me*,* ‘When can I be discharged again*,* when do I no longer have to come here?’[…] (Nurse*,* _P02_03*,* Pos. 5)*

Retrospective reflections on the decision to start dialysis may also arise during these conversations:

*Sometimes they also say*,* ‘If I had known that*,* I might not have started dialysis.' (Nurse*,* P01_04*,* Pos. 7)*

These conversations arise within ongoing clinical encounters rather than as part of a structured or scheduled discussion. They may be initiated by patients themselves or emerge when signs of burden become apparent and are addressed during consultations:

*Well*,* you certainly only talk to a patient about it if you notice that something is troubling them or if*,* as I said*,* they express pain or simply don’t want to continue. That’s when we start a conversation with the patient. (Physician*,* P01_06*,* Pos. 5)*

#### Therapy goal adjustments

HCPs report that patients sometimes wish to discontinue dialysis. These preferences are considered in relation to disease progression and treatment circumstances:

*If patients then say they don’t want to continue*,* I usually start a conversation about the possibility of stopping dialysis. (Nurse*,* P01_01*,* Pos. 21)*

*This is raised by some relatives*,* but also sometimes by patients*,* who say quite clearly*,* ‘I don’t want to carry on like this*,* I can’t take it anymore*,* my body won’t let me. I’m too weak*,* too tired*,*’ and then you give them advice on what they can do. (Nurse*,* P02_05*,* Pos. 11)*

HCPs emphasize the importance of early discussions about dialysis initiation, ideally already during hospitalization, to allow patients to make informed decisions and avoid starting dialysis when it may no longer align with overall therapy goals:

*That’s what I’d like to see most of all. I want the hospital already to actually consider whether dialysis is necessary […]. What we end up with are people who are truly desperate*,* and it becomes even harder for them once they start thinking*,* ‘I don’t want this’. (Physician*,* P01_06*,* Pos. 21)*

Dialysis is perceived as a life-prolonging measure. Decisions about continuing or discontinuing treatment therefore have direct consequences for patients’ lives:

*And also*,* I mean*,* in the end*,* dialysis usually just keeps patients alive*,* but they still have a serious underlying disease or cancer*,* whatever*,* right*,* and they’re already suffering*,* right? (Nurse*,* P01_01*,* Pos. 29)*

*That’s difficult because*,* unfortunately*,* in our case*,* therapy is such that in most cases*,* if you get therapy*,* you stay alive*,* and if you don’t get therapy*,* you die. (Nurse*,* P03_01*,* Pos. 27)*

Other concerns of HCPs are about possible symptoms and complications that could arise from discontinuing dialysis, such as dyspnea or fluid retention. They also take into consideration the need for follow-up care by GPs or palliative care services in the context of integrated care in order to ensure continuity of care. The goal is a symptom-controlled and dignified dying process:

*And you can’t just stop dialysis at that point*,* can you? You have to keep supporting them*,* because they’ve already taken that step*,* right? […] It’s not like you can just leave patients to their own fate and [let them] go home*,* stop dialysis*,* and not do anything else. That’s not how it works*,* right? (Nurse*,* P01_01*,* Pos. 33)*

#### Team communication

Team communication about patients’ palliative care needs takes place in various ways in practice. These interactions range from unstructured, adhoc conversations between staff to formal discussions in scheduled meetings. Unstructured communication often occurs during shifts or breaks, as well as in brief preliminary discussions before rounds. Nurses exchange observations and express needs with each other and, if necessary, discuss them with physicians:

*[…] there is usually a quiet period between meals when we discuss the patients. Not so much during treatment*,* though. As I said*,* everyone is very focused on the dialysis work. During the breaks*,* however*,* we like to talk about it. (Nurse*,* P01_05*,* Pos. 11)*

*We don’t really do this in a very institutionalized way*,* so we don’t have a fixed team meeting*,* but it’s common practice to discuss these issues when preparing for rounds*,* as we visit patients three times a week*,* and always hold a short team meeting with the nurses on the ward before rounds*,* which is when these topics come up. (Physician*,* P02_01*,* Pos. 9)*

Regular team meetings lead to structured discussions in some teams, for example during weekly team meetings, monthly management meetings, or during daily handoff, sometimes separated by professional groups:

*Otherwise*,* we also discuss things like this during handoffs*,* such as if we notice that a patient has become very depressed or is going through a depressive phase*,* in which case we need to keep a closer eye on them. (Nurse*,* P03_02*,* Pos. 19)*

*The doctors have their meetings once a week. That’s when they discuss various patients and issues*,* as well as upcoming tasks and tests. I’m not really sure. But we*,* as a nursing team*,* don’t really do that. (Nurse*,* P01_02*,* Pos. 23)*

Some teams also have monthly meetings with the senior nurse to discuss any issues or special concerns relating to patients. These meetings can also address topics such as adjustments to dialysis or its termination and are scheduled to allow detailed discussions:

*And then we also have a day when our head nurse comes*,* which happens once a month […] and we discuss any issues with patients […]. But the meetings are really structured*,* especially when it comes to ending dialysis […]. We have plenty of time for that. So our meeting lasts over two hours*,* and we have enough time to not rush through it. (Physician*,* P01_06*,* Pos. 9)*

#### Collaboration with external actors

For patients with the wish to discontinue dialysis the GP is often involved. They coordinate palliative home care, treat symptoms, involving relatives in the process. In particularly challenging situations, such as existing complex symptoms, a palliative care unit is also consulted to ensure close monitoring. However, direct communication between palliative care physicians and nephrologists is rare:

*So*,* we don’t really have anything to do with palliative care*,* right? So*,* we just make sure that we inform the GP so that he can take care of the palliative care*,* or […] that the hospital takes care of it*,* right*,* so that palliative care is provided*,* right? Exactly. (Nurse*,* P01_01*,* Pos. 23)*

Psychological support can also be called upon to assist patients and their relatives:

*[…] or perhaps seek external support from a psychotherapist who can simply take another look at the situation […]. Because sometimes it’s not just the dialysis*,* but other things that are behind it. (Nurse*,* P03_02*,* Pos. 15)*

#### MINI implementation

The MINI, including its training component, was reported to raise awareness of palliative care issues, but it was not perceived as leading to concrete or lasting changes. In practice, the identification of palliative needs remained largely unstructured and occurred as situations arose:

*Well*,* I think there’s greater awareness of palliative care issues and the real concerns patients face at the end-of-life. Yes*,* so*,* sensitivity to this [is increased]. (Physician*,* P02_01*,* Pos. 23)*

*Probably right after it happens*,* at least at first*,* but then you just fall back into your daily routine*,* I don’t know. Yeah*,* so I can’t really say that it’s changed anything*,* can I? (Nurse*,* P01_02*,* Pos. 55)*

The Surprise Question was described as simple and feasible to apply. At the same time, participants emphasized that the question is not a fully reliable prognostic instrument, as disease trajectories can be unpredictable. HCPs reported that they were able to answer it more confidently when they had known patients for an extended period and observed a continuing decline in their general condition:

*Especially when you’ve gotten to know the patients over time and you can really see that their overall condition has been steadily declining*,* you could honestly answer with a clear ‘no’ that you’re not at all surprised. So*,* I think that [meaning the Surprise Question] could definitely be used.* (Medical Assistant, P02_04, Pos. 41)

The SPICT criteria were perceived as fundamentally useful, but their practical application in day-to-day practice remained limited. Participants reported that the large number of criteria was difficult to remember and time-consuming, so they therefore rarely used them:

*[…] the SPICT criteria are twofold. In principle*,* they’re easy to apply. But because there are so many of them*,* you can’t keep them all in your head. At least I couldn’t. (Physician*,* P02_01*,* Pos. 17)*

Participants reported that they found the communication tools (NURSE, SPIKES) helpful for addressing emotions and structuring conversations with patients. They noted that the tools were often applied intuitively or based on previous experience, while the pocket cards themselves were used only occasionally. The tools confirmed existing communication practices, as HCPs were already communicative and had previously addressed patients’ concerns, rather than fundamentally changing communication behaviors:

*What I really liked*,* […] is this NURSE for dealing with emotions. I thought it was great*,* both in terms of how it’s designed and how it can be applied. I haven’t used SPIKES that often; I don’t even really know exactly what the reason may be*,* or perhaps I should put it another direction*,* I might have just used it intuitively*,* based upon what I had learnt in my studies […] (Physician*,* P02_01*,* Pos. 17)*

**Table 5:** Barriers and facilitators as reported in the interviews to the implementation of the MINI and the integration of palliative care, structured according to CFIR domains

CFIR Domain	Key barriers	Key facilitators
Innovation	SQ was perceived as difficult to apply in unfamiliar or unstable patients. SPICT required preparation and familiarization. Some written materials were considered less suitable for outpatient care.	Existing evidence on SQ and SPICT increased confidence in the intervention. The intervention was perceived as supporting clinical reflection and decision-making and could be adapted to local workflows. Concise materials (e.g., pocket cards or structured guides) were considered helpful for implementation.
Inner Setting	Limited time, staffing constraints, competing priorities, and lack of privacy hindered palliative care discussions. Limited nephrology-specific palliative care knowledge and a treatment-oriented culture reduced confidence and engagement. Communication gaps and fragmented responsibilities affected continuity of care.	Training and refresher sessions were perceived as to be helpful in improving knowledge and confidence. Structured communication, interdisciplinary collaboration, leadership support, and regular case discussions could facilitate implementation. Protected time and clear responsibilities might support identification of palliative care needs.
Outer Setting	Limited availability of palliative care services, fragmented outpatient–inpatient structures, delayed access to specialist services, and insufficient reimbursement created barriers. Financial and institutional incentives often favored continuation of dialysis treatment.	Collaboration with GPs and palliative services could improve coordination of care. Increased visibility and awareness of palliative care in nephrology might support integration into routine care.
Individuals	HCPs: Limited knowledge or training in palliative care, emotional burden, and uncertainty in end-of-life communication. Patients: Fear, reluctance, or changing preferences regarding palliative care and treatment continuation. Relatives: Reservations toward palliative care and emotional burden in end-of-life situations.	HCPs: Communication experience, patient-centered motivation, and willingness to collaborate were perceived as beneficial. Patients: Ability to express informed preferences and cognitive capacity would support decision-making. Relatives: Involving and supporting relatives to help them understand and accept palliative care or the adjustment of life-prolonging treatments

## Discussion

Our study aim was to explore HCPs’ perspectives on palliative care integration in outpatient nephrology, to assess knowledge and confidence in identifying and supporting seriously ill patients, and to identify barriers and facilitators to implementing structured palliative care approaches.

Our findings indicate that HCP in outpatient nephrology recognize the importance of integrating palliative care to address the complex needs of patients with advanced kidney disease. They see the benefits of early palliative integration into nephrology primarily as a more patient-centered care, including management of physical and psychological symptoms. Yet, only about half of the participants reported feeling confident in identifying and caring for patients in their last year of life as well as familiarity with criteria used to recognize such patients. These findings suggest that knowledge and confidence regarding the identification of patients approaching the end-of-life remain limited among a considerable proportion of HCP. Insights from the qualitative interviews support the interpretation that timely identification of patients with potential palliative care needs is hindered by uncertainty. The qualitative interviews indicated that palliative care needs were often addressed reactively, with conversations arising in response to symptoms, changes in patients’ condition, or signs of burden rather than being systematically integrated into routine care. In addition, the often fluctuating and unpredictable clinical condition of patients with advanced kidney disease was perceived as making it difficult to determine the appropriate timing for initiating palliative care, which is consistent with previous research [38]. Interview data indicated that while the MINI as a full framework was not routinely used, the SQ was readily adopted. The SPICT tool was perceived as useful, but its use remained limited in everyday routine, reflecting time pressure and the complexity of the criteria as hindering factors also found in a prior study [27]. However, HCP reported increased awareness of patients’ palliative needs and greater confidence in addressing end-of-life issues. Despite these perceived benefits, sustained use of the MINI in routine practice remained limited, suggesting that individual factors such as awareness and confidence alone may not be sufficient to shift palliative care from a predominantly reactive approach, in which needs are addressed in response to emerging concerns or changes in patients’ condition, towards more systematic and proactive integration into routine care. This is consistent with previous qualitative research highlighting challenges in providing timely and proactive end-of-life care within nephrology and the need for greater integration of palliative care into routine clinical practice [39].

Few participants addressed patients’ preferences regarding place of care and place of death. This is in line with a previous study, showing that only a third of nephrology nurses communicate with their patients about their preferred place of death [40]. Dialysis patients with ESRD may particularly benefit from discussions about their preferred place of care and death as this is strongly recommended for patients with chronic diseases and multimorbidity, since they are more likely to die in hospital than at home [41–44].

More than half of our participants communicate generally about advanced directives; however, few advised and assisted patients in drafting such a directive. A study found that discussing Advance Care Planning (ACP) with a goal-orientated approach instead of a problem-oriented approach may be helpful for HCP to engage in this discussion [41]. Furthermore, knowledge gaps and uncertainties in legal and ethically complex areas such as termination of life-prolonging measures exist, which are common in other medical fields or in palliative care itself [45–47].

About a quarter of our participants see the responsibility of end-of-life conversation on the part of the specialist or GP, respectively. About a third esteem palliative care services and only a few see hospital physicians as responsible for end-of-life communication. Studies suggest that non-cancer patients receive less palliative care and have fewer conversations about the severity of the disease: a reason can be that cancer patients discuss these topics with their treating specialist, whereas non-cancer patients have these discussions with their treating GP, mostly [48]. Considering this, nephrological patients are seen rather more often by a specialist, e.g. a nephrologist, than by the GP, indicating that specialists play a central role in decision-making and may represent an important professional group for strengthening the integration of palliative care [49, 50]. However, specifically, most nephrology patients report that the nephrologist did not communicate with them about the prognosis [1]. Opportunities for proactive conversations with patients and relatives are limited, both in terms of dedicated time and private spaces, which hinders not only the systematic identification of palliative needs but also the proactive integration of palliative care into routine management [51]. A primarily therapy-oriented culture can make palliative integration challenging when priority is given to the actual medical treatment, e.g. dialysis and financial or structural incentives that may favor continuation of dialysis [52]. Facilitating factors were clear role definitions, regular and structured team communication, which relies not only on internal team communication but also on collaboration with external healthcare providers. Few participants reported on external communication with GPs or palliative care services, suggesting that integration in interprofessional networks and continuity of care are limited. However, insights from interviews indicate that when such communication occurs, GPs often serve as a hub to palliative care services, supporting patient transitions and facilitating ongoing care. HCPs report their wishes for a smooth transition when dialysis is terminated, e.g. information about potential symptoms and relief of symptom burden, which is also described by a previous study [53]. Still, direct communication pathways and routine interfaces with external services remained limited due to cross-sector separation, low availability of local palliative care resources and unclear responsibilities.

### Strengths and limitations

A key strength of this study is its multi-method design, which allowed triangulation of quantitative and qualitative data and provided complementary insights into current practice and experiences with a structured palliative care approach. Another strength is its focus on the outpatient nephrology setting, which provides an important context for examining how palliative care needs are recognized and addressed within ongoing care for patients with complex and unpredictable kidney disease trajectories.

Limitations include the use of convenience sampling, which may limit representativeness. Also, the possibility of a nonresponsive bias due to the inability to determine a precise response rate (based on the study design and data anonymization) cannot be excluded. Another limitation is the low response rate for the post-survey, which therefore could not be included in this manuscript as well as possible data loss due to dichotomization of variables. The self-reported nature of survey data may also have introduced social desirability bias and as not all items of the questionnaire have been validated in other studies, these results must be interpreted with caution. Since there is no validated instrument for this special patient group, the questionnaire should be tested and validated in further research. The sample was predominantly female and included fewer nephrologists than other professional groups, which may have limited the range of perspectives represented. The interview duration may have limited opportunities to explore some topics in greater depth; however, it was sufficient to address the research question. Telephone interviews may have limited the capture of non-verbal cues and spontaneous interaction but enabled flexible participation and were appropriate given the practical circumstances of the study.

### Conclusions

This exploratory study provides insights into how palliative care is currently recognized and addressed in outpatient nephrology and highlights barriers and facilitators to its integration. Healthcare professionals valued early palliative care, but confidence in identifying patients approaching the end of life and routine communication about prognosis, preferences, and advance care planning were inconsistent. Palliative care needs were often addressed reactively as concerns or changes in patients’ condition arose, rather than being proactively integrated into routine care. The MINI and its individual components were perceived as helpful in increasing awareness and confidence, although the full approach was not consistently used in everyday practice.

These findings suggest that structured and low-threshold approaches such as MINI may support a more systematic and proactive integration of palliative care into routine nephrology care. However, their use also depends on organizational conditions, including protected time, regular and structured team communication, clear responsibilities, and established communication pathways with general practitioners and palliative care services.

Further research should examine the feasibility and longer-term effects of structured approaches such as MINI in routine outpatient nephrology, including differences between professional groups and potential roles for dedicated palliative care responsibilities within nephrology teams. Patient and relatives’ perspectives should also be investigated to inform the development of approaches that are responsive to their needs and preferences.

## Supplementary Information

Below is the link to the electronic supplementary material.

Supplementary Material 1

Supplementary Material 2

Supplementary Material 3

## Data Availability

Data is available on reasonable request to the corresponding author melanie.joshi@uk-koeln.de.
