# Supplementary material for: Early integration of palliative care in outpatient nephrology: exploring healthcare professionals’ knowledge, attitudes, and communication practices – an exploratory multi-methods study
Source: BMC Nephrol. 2026 Oct 9;27:603. doi: 10.1186/s12882-026-05409-8 (PMC13654204; doi:10.1186/s12882-026-05409-8)
Supplement: Supplementary file 1 [file 12882_2026_5409_MOESM1_ESM.pdf]

	**A. Introduction**
1.	What do you believe constitutes good care for people with severe kidney disease?
2.	What are the topics that patients or their relatives have brought up with you when it comes to their serious illness?<br>o How do you deal with this?
3.	What opportunities are there in your team to discuss patient needs?<br>o What obstacles do you face when discussing patient needs within the team?<br>o Do you recall examples of situations in which communication about patient needs was particularly effective?<br>o What support or resources would help you facilitate this exchange?
	**B. Palliative care vs. nephrology**
4.	What experience have you had with integrating palliative care into nephrology?<br>o How does communication with palliative care specialists currently work?<br>o What support or resources would help you facilitate this exchange?
5.	*If not mentioned above:* What challenges do you see in integrating palliative care into nephrology*?*<br>o What can help to counteract the challenges (facilitating factors)?
6.	How do patients and their relatives benefit from the integration of palliative care into nephrology?<br>Do you see any disadvantages to this?
	**C. MINI-Intervention**
7.	To what extent did the intervention help you identify palliative care needs in patients?<br>o How helpful did you find the application of SQ?<br>o How helpful did you find the application of SPICT?<br>o How helpful did you find the use of the conversation guide for patients?<br>o What were your reasons for not using the intervention?
8.	To what extent has the training/intervention changed your perspective and your approach to patients? (e.g., communication)
9.	To what extent has the training and its subsequent application changed anything within the team in practice?<br>o E.g. in relation to topics such as “dying,” “death,” “grief,” and “serious illness?
10.	Will you continue to use the intervention after the study has ended?<br>o What would be necessary for you to continue using the intervention (SQ, SPICT) in your everyday care practice in the future?
	**D. Conclusion**
11.	What would you like to see in terms of better support and care for people with severe kidney disease and their relatives?
12.	Are there any points that have not yet been addressed but that you would like to share?
