# Supplementary material for: Early integration of palliative care in outpatient nephrology: exploring healthcare professionals’ knowledge, attitudes, and communication practices – an exploratory multi-methods study
Source: BMC Nephrol. 2026 Oct 9;27:603. doi: 10.1186/s12882-026-05409-8 (PMC13654204; doi:10.1186/s12882-026-05409-8)
Supplement: Supplementary file 2 [file 12882_2026_5409_MOESM2_ESM.pdf]

Description of the coding framework

Main category	Subcategory	Description
**Characteristics of quality care for seriously ill patients**	—	Statements describing characteristics of high-quality care for seriously ill patients in outpatient nephrology.
**Palliative care in nephrology**	Specific conversations / conversation topics	Statements concerning conversations about fears, wishes, meaning, life-limiting illness, death, dying and grief.
	Exchange about patient needs	Statements concerning how and when patient needs are discussed within the care team, including routines, opportunities and challenges.
	Collaboration with external care providers	Statements concerning collaboration and communication with external palliative care services and other healthcare providers.
	Therapy goal change	Statements concerning changes in treatment goals, including decisions regarding discontinuation of dialysis and transition towards palliative care.
	Advance care planning	Statements concerning advance care planning and patients' preferences for future care.
	Patient characteristics	Statements concerning patient characteristics perceived as relevant to palliative care, including age, multimorbidity, cancer, psychosocial burden and changes in the patient population.

Main category	Subcategory	Description
**Barriers to palliative care**	Financial aspects	Statements describing financial or reimbursement-related barriers to palliative care.
	Barriers to specific communication	Statements describing factors that make conversations about death, dying, grief or other sensitive topics difficult.
**Facilitators of palliative care**	—	Statements describing factors that facilitate the integration of palliative care, including supportive structures, collaboration, training and education.
	Development of palliative care structures/education	Statements concerning suggestions and opportunities for developing palliative care structures, responsibilities and educational activities.
**MINI approach**	—	Statements concerning participants' experiences and perceptions of the MINI approach.
	Perceived (non-)effect	Statements concerning perceived effects or lack of effects of MINI and its components.
	Implementation and acceptance	Statements concerning the introduction, use, acceptance and integration of MINI into routine care.
