# Supplementary material for: Early integration of palliative care in outpatient nephrology: exploring healthcare professionals’ knowledge, attitudes, and communication practices – an exploratory multi-methods study
Source: BMC Nephrol. 2026 Oct 9;27:603. doi: 10.1186/s12882-026-05409-8 (PMC13654204; doi:10.1186/s12882-026-05409-8)
Supplement: Supplementary file 3 [file 12882_2026_5409_MOESM3_ESM.pdf]

CFIR Domain	Barriers	Facilitators
Innovation (MINI-Intervention)		
Evidence-Base	n.a.	• International prior work and existing studies increased confidence in the intervention.<br><br>*„[…] back then I had this American colleague, […], send me the lecture. I found it interesting what they were already dealing with back then."* (Interview P01_06)<br><br>*„[…] I know there are also studies showing that it is very significant […] [referring to the Surprise Question] […]"* (Interview P01_06)
Relative Advantage	n.a.	• The intervention was perceived as helpful in supporting reflection and decision-making in situations of clinical deterioration or increasing complexity.<br><br>*"So if the patients are already somewhat along and then maybe another illness comes into play or you notice, you know, the patient is getting worse and the general condition, then that's [referring to the Surprise Question] actually a good thing, right?"* (Interview_P01_01)
Adaptability	n.a.	• The intervention was perceived as adaptable to local practice needs through selective use and tailoring of content and questions.<br>*"[…] a kind of questionnaire, that could be for patients but specifically tailored to our patients, right?"* (Interview_P01_03)

Complexity	• Difficult to use with new or very ill patients at initial encounters.<br><br>*"Since we haven't really worked with it, you know, what's difficult is right at the beginning, when the patients start here with us and we think, especially if they are too old or very sick, I can't just take the booklet on the first day and ask my questions, because I don't know the patient at all, you know?" (Interview_P01_01)*<br><br>• Requires substantial preparation and familiarization before use of the intervention materials.<br><br>*"No, it's really one of those things where you say, oh, I have to read through everything first, this and that, yes, it must certainly cover a lot, but it's not like you just walk around with the sheet." (Interview_P01_04)*	n.a.
Design Quality	• Some content of the material was not suitable for an outpatient setting.<br><br>"There's a bit in the booklet, there are things in it, we are an outpatient practice, right, not inpatient, so some things are not suitable." *(Interview_P01_01)*<br><br>• Preference for verbal interaction over written materials.<br><br>"With the guidelines. [...] I'd rather just talk to the patient. I don't really want to hand them a written document just yet, right?" *(Interview_P01_04)*	• Concise materials (e.g., flyers, structured questionnaires) could support new staff and ease implementation<br><br>*"I think it would be helpful to have flyers like the ones you received during the training session available on the ward as well. [...] I imagine this would be helpful for colleagues who, for example, are new to the field and have not yet had any experience with this particular topic." (Interview P02_04)*<br><br>• A comprehensive guide was valued for reference and orientation.

		"So, what they said, a patient questionnaire with given criteria, that would definitely be good / like a big guide, so you can read into the whole thing, that would definitely be great." *(Interview_P01_05)*
Inner setting		
Access to Knowledge and Information	• A lack of knowledge and training among healthcare professionals leads to uncertainty and reluctance to initiate discussions about aspects of palliative care.<br><br>*"I think, first, ignorance, also among the nephrologists on this topic, I would also say fear of addressing it themselves, because of lack of knowledge and not being well trained or not trained at all. Yes, that is certainly the main reason, so there is probably also a lack of willingness to address it themselves, but I would rather see that as a consequence because one does not feel competent in it." (Interview P02_01)*<br><br>• Limited nephrology-specific information on palliative care undermines confidence and preparedness.<br>• Limited experience and training in dealing with severe illness and death.<br><br>*"[…] Especially from our young colleagues, some of whom are not even from nursing, but medical assistants, dental assistants who retrained, who have never seen a deceased person or dealt with it at all." (Interview_P02_05)*	• Targeted training and educational sessions increase staff knowledge and skills.<br><br>*"So I would need more information material. It would help me if I were trained, just for nephrology patients, palliative care for nephrology patients." (Interview_P02_03)*<br><br>• Refresher courses and ongoing education help maintain competency, especially for new staff.<br>*"[…] For example, another training session, if one had the option again. Especially because we now have many new colleagues, […] I would find it really good to refresh it again, yes."*<br><br>• Staff with prior communication training can support implementation and mentoring.<br><br>*"I think it would need, like we did now, the nurses who have a bit of knowledge in communication." (Interview P01_06)*

Structural Characteristics	• Dependence on external providers (e.g., GPs) for prescribing medication or home visits limits ability to address patient needs.<br>• Limited time and competing responsibilities reduce opportunities to recognize patients' palliative needs.<br><br>*"[...] That you have time for him [the patient], that you listen and don't have to leave immediately because another alarm rings or because I have to go to another patient." (Interview_P02_03)*<br><br>• Restricted access to palliative documentation (e.g., specialist notes) hampers comprehensive understanding of patient status.<br><br>*"For us, it is only documented in palliative treatment, possibly medications as well, but not discharge summaries from the palliative physician—I have no access to that." (Interview_P02_03)*	• Clear roles, responsibilities, and dedicated points of contact support coordination.<br><br>*"[...] It would of course be good if someone takes it firmly in hand, you know, a fixed contact person here in our center." (Interview_P01_05)*<br><br>• Regular, structured conversations about care goals and patient status support identification of palliative needs.<br><br>*"[...] They also had nurses who conducted regular conversations about therapy goals and the current state of wellbeing." (Interview P01_06)*<br><br>• Protected time for patient interactions allows recognition of patients' palliative needs.<br><br>"[...] But if I turn to a patient and he opens up to me and explains his distress, then I must also have time for him and not let anything else disturb me." *(Interview_P02_03)*
Relational Connections	• Frequent changes in responsible physicians disrupt continuity of collaboration.<br>• Effective communication depends on individual colleagues' engagement and support.<br><br>*"[...] We always try to talk to each other, say, wouldn't it be good to involve [palliative care] now, and yes, we also bring*	• Close cooperation and regular exchanges between professional groups strengthen information sharing.<br><br>*"Despite everything, I always have the impression that I can approach one of our doctors, who then also take care of the problem, yes, who then deal with it with me." (Interview_P02_03, Pos. 9)*

	it up to the doctors, although not all doctors are immediately pleased. They are a bit blocked at first [...]." (Interview_P01_05, Pos. 13)<br>*"And in the end, you also have to see what consequence it has, right? You can discuss it with the doctors, but it is always difficult." (Interview_P01_02, Pos. 67)*	• Clarification of roles and responsibilities ensures coordinated patient care and follow-up.<br>*"[...] Because then it is also said who sees the patient next, who addresses what again, who possibly takes care of contact with the GP." (Interview P01_06)*<br>• Accessible leadership and experienced staff provide guidance and support for complex patient situations.<br>*"I find our head of nursing has an open ear, you can go to her with a problem and maybe get a quick piece of advice, because she might have a bit more experience than one yourself." (Interview_P03_02, Pos. 19)*
Communications	• Communication deficits among professionals limit coordinated care.	• Regular, scheduled meetings provide dedicated time for thorough discussions about patient palliative care needs.<br>*"We actually talk all the time during the shift, even during breaks. So it's constant communication. How is the patient? How can we do it better, and basically always in connection with a doctor." (Interview_P01_03)*<br>• Ongoing informal communication during shifts and breaks maintains team awareness.<br>*"More collaboration with doctors, with us, so that they also listen to our questions or what we say, because we see the patients much longer than our doctors or doctors in general, not only with us but overall, so that there is more communication."* (Interview_P02_05, Pos. 67)

		• Collaborative exchanges with physicians ensure staff input is considered.<br>• Systematic documentation of patient goals and follow-up discussions supports continuity of care.<br>• Team discussions or case reviews help align understanding across staff<br><br>*"[...] Conversations also among colleagues and possibly on the wards where a patient is, so that colleagues maybe discuss cases among themselves. I can also very well imagine this with the treating doctor, maybe, so that it's a team construct, so that colleagues on the ward are all on the same knowledge level about the patient, how the patient feels, so case discussions would certainly make sense in the future." (Interview_P02_02, Pos. 43)*
Culture	• Reluctance toward palliative care<br>• Predominant treatment-oriented focus in clinical practice.<br>• Reduced attention and responsiveness to patients' needs.<br><br>*"[...] And then the doctor says, 'no, we have to,' and I say, 'what do the relatives say? He has sons.' Then he called and said, 'no, no, we should do everything.' Well, we dialyzed him." (Interview_P02_05, Pos. 19)*<br><br>*"Also understanding of many things too, because sometimes I feel that they don't really notice it, don't see it, the training is not there, yes? Many also have empathy and they know what it is. And some just partly see it as a job,*	• Openness and sensitivity toward palliative care among staff.<br>• Awareness and proactive consideration of patients' concerns and palliative needs.<br>• Team support and shared reflection when patients die or experience deterioration.<br><br>*"[...] In our practice, we were never the ones who said 'dialysis at any cost.' We were not like that at all; we always saw it in the interest of the patient." (Interview P01_06, Pos. 31)*

	which unfortunately happens much more nowadays than one thinks." (Interview_P02_05, Pos. 25)<br><br>*"I think it's a generational conflict [...] among the nursing staff. I have observed that our younger colleagues, who are not doing worse work, for God's sake, but don't really have the sense for it and maybe don't always have the patience to give the patient the attention he needs." (Interview_P02_03)*	*"Actually, everyone is open. Especially with the topic of palliative care, which was also brought from the [training institution], which we also implemented here with me, yes, they have all become more open to address it more and think more about such things, about palliative care." (Interview_P01_05, Pos. 69)*<br><br>*"[...] That there is simply more sensitivity and also involving palliative medicine, i.e., the palliative doctors, so that there are colleagues who really provide helpful support." (Interview P01_06, Pos. 31)*
Tension for Change	n.a.	• Awareness that palliative care is important for the team.<br>• Consideration of patients' presumed wishes and needs, including those with cognitive impairment.<br>• Desire to offer patients more dignified care options.<br>• Recognition of potential to improve care quality and address unmet needs.<br><br>*"And I think a bridge really has to be built so that somehow the patient can know that it can also be done differently, more humanely, I would just say." (Interview_P02_02, Pos. 9)*<br><br>*"So I think there is room for improvement in all areas." (Interview_P02_02, Pos. 17)*<br><br>*"We see a lot of this with our dying patients as well; we have many more cases where patients are admitted to us and then*

			die very quickly, and I believe we could do something to address this." (Interview P01_06, Pos. 23)
Relative Priority	• Competing tasks, workload, and ongoing trainings limit time to engage with new initiatives.<br>• Insufficient time to implement or integrate new practices.<br>• Limited personal focus or engagement with the topic due to other priorities.<br><br>*"I am multitasking and for the past two years I have been almost overwhelmed here with different tasks and training. And what needs to be implemented or applied requires time until it really works. And therefore, personally, I have not dealt with the topic." (Interview_P03_01_überarbeitet, Pos. 35)"*	n.a.	
Available Resources	• Limited time per patient restricts in-depth palliative care discussions.<br>• Staffing constraints and multitasking limit opportunities to address palliative care needs.<br>• Shared dialysis rooms reduce privacy and hinder sensitive palliative care conversations.<br><br>*"Yes. It is difficult when we first take over dialysis here, and maybe the time is also missing to bring it up directly." (Interview_P01_03, Pos. 41)*		• Sufficient time and staffing to identify and address patients' palliative care needs.<br><br>*"Time. Time for the patient." (Interview_P01_03, Pos. 69)*<br><br>*"More time. More time for the patients. So due to the nursing shortage it is a bit limited, but more time would be nice. Simply to talk in much more detail with the people or the relatives." (Interview_P03_02, Pos. 35)*

	"Because you might not have the chance to talk to the patient as you would like, because you are understaffed and you know, okay, I don't have enough time for this patient and I have to move on to care for the next patient." (Interview_P03_02, Pos. 31)<br><br>*"Even just for a word in between, there is little time. When connecting or disconnecting, you get into conversation, but it is only a few minutes. Really in-depth is more difficult, and they don't sit in single rooms with us but in large groups." (Interview_P01_02, Pos. 15)*	
Outer Setting		
Local Attitudes	• Negative perceptions of palliative care creating fear or reluctance<br><br>*"So I know from Clinic XY, at that time it had ten beds, which was very few for such a large clinic, and therefore it is still in people's minds: palliative terminal ward, and yes, many are scared of it."“ (Interview_P02_05, Pos. 35)*	n.a.
Local Conditions	• Limited local availability of palliative care services<br><br>*"I unfortunately heard here in [state XY] that there are practices where it is not an issue at all, where from the nursing side it is not allowed to discuss stopping dialysis." (Interview P01_06, Pos. 31)*	• Increased availability of local palliative care resources and services<br><br>*"This was not given before, because the pathways that exist today are structured like this; sure, palliative care existed before, but not to this extent. There were not so many contact points and opportunities. Today we have palliative nursing service. So today you feel, I feel, there are much, much more*

	"For that, we actually have too few palliative wards here."#0 (Interview_P02_05, Pos. 31): Mangel an Palliativmedizin-Betten<br><br>*"So we don't have a palliative team directly on site." (Interview_P01_03, Pos. 27)*<br><br>*"Because there is a shortage everywhere, and to bring someone or organize it, I mean, it takes too long, yes, for some it is then too late to simply improve home care a bit." (Interview_P01_04, Pos. 71)*	*possibilities, a much closer network you can rely on." (Interview_P02_02, Pos)*
Financing	• Lack of financial incentives or reimbursement reduces motivation to conduct palliative care conversations.<br>• Dialysis therapy is resource- and time-intensive, insufficiently reimbursed, and associated with high workload pressure.<br>• Bureaucratic hurdles and limited reimbursement restrict access to palliative care.<br><br>*"I can't tell you now, maybe the health insurance doesn't cover it, or I don't know, it's a bureaucratic mistake or not a mistake, but a bureaucratic hurdle, I think, that they just can't come directly. I think that's very unfortunate. I think they themselves can't do anything about it or don't want to / they do want to help, but the bureaucracy says no, especially the health insurance, it doesn't work."#00:09:59-5# (Interview_P03_02, Pos. 25)*	• Adequate reimbursement or financial incentives for providing palliative care would encourage engagement.<br>• Desire for equitable compensation regardless of the type of care provided (dialysis, palliative, or combined interventions).<br>• Increased funding for ambulatory practices to support comprehensive patient care.<br><br>*"I have to say it clearly: reimbursement. I'll put it broadly, the wish would actually be that I get the same amount of money regardless of how I treat the patient, whether I treat them palliatively without, for example, dialysis or instrumental medicine, or if I carry out instrumental measures and at the same time maybe palliative measures like conversation support, pain therapy, etc., that would of course be the ultimate wish." (Interview P02_01, Pos. 29)*

	"Whereas certainly one point is that it is not reimbursed, which is of course somehow stupid. I mean such a conversation, suppose it lasts an hour, we do it anyway, but I think it would definitely be an incentive if there was a reimbursement situation." (Interview P02_01, Pos. 15)	*"Exactly, very simply. I wish that more money comes into the outpatient practice." (Interview_P03_01_überarbeitet, Pos. 57)*
Policies and Laws	• Separation between outpatient and inpatient sectors creates gaps in continuity<br>• Access to specialized palliative home care requires approval by health insurance, delaying timely support.<br><br>*"So it is, you know, and the patients want to go to the hospital but are discharged again because the indication was not really given, but the patient is actually afraid to go home because he thinks he can't manage at home, right? We have that very often, that they are just sent home, right? The uncertainty, you know?" (Interview_P01_01, Pos. 63)*	N.a.
Partnerships and Connections	• Limited direct interaction with palliative care specialists; nephrology teams mainly act as intermediaries to primary care physicians.<br>• Access to specialized palliative services (e.g., specialized palliative home care) can be delayed, especially in urgent situations or outside regular hours.<br>• Low visibility of palliative care within professional networks and conferences reduces awareness and integration opportunities.	• Nephrology team collaborates with primary care physicians and palliative care teams to coordinate care.<br>• Structured involvement of palliative services (home-based or hospital-based) supports continuity and shared responsibility.<br><br>*"That everything is then taken care of via the GP or the palliative ward or palliative nurses, right, so that you are not standing alone, right? So actually this is often picked up like that with us, right?"* (Interview_P01_01, Pos. 21)

"Exactly, so you can summarize: we have little to no contact with palliative specialists, but we are the intermediaries to the GPs." (Interview P02_01)

"So we actually don't deal with palliative care at all, right? We just take care to inform the GP so that he handles palliative care, or our doctor XY, who was at hospital XY, so that through the hospital, the palliative care is provided, right?" (Interview_P01_01)

"That lies in the hands of our doctors, who are in close exchange with the GP." (Interview_P02_03, Pos. 31)

"But of course we also have patients who are very connected through SAPV, and if it really doesn't work anymore, it is then stopped, and so far I have only heard good things; we just had a patient recently who unfortunately did not make it into SAPV because it was the weekend, she was very ill, you could see she was already very pre-final, I would say, and SAPV does not admit on weekends [...] Yes, there was an emergency number, but this emergency number is apparently only for existing patients who are already in this service." (Interview_P03_02, Pos. 25)

"Visibility in the network is missing (conferences): There simply has to be more / I think we are, or the topic of palliative medicine is simply not present enough at conferences." (Interview P01_06, Pos. 43)

External Pressure	• Industrial providers may influence staff to prioritize treatment over addressing palliative care needs.<br>• Weekly reimbursement models create pressure to continue dialysis, influencing clinical decisions.<br><br>*"Then, basically industrial / partly industrial providers, so that the nursing staff says the topic must not be addressed at all. And I find that really sad—such a thing must not exist, that dialysis is continued to get weekly flat rates, but unfortunately you hear about it." (Interview P01_06, Pos. 43)*	• Increased visibility and awareness of palliative care in nephrology encourages its consideration and integration into practice.<br><br>*"I think this [referring to palliative care] just needs to be more widely recognized among nephrologists."* (Interview P01_06)
Individual		
Patients	• Patients' preferences and decisions regarding their care can change over time (e.g., continuation or cessation of dialysis).<br>• Fear or hesitation may lead to reservations toward palliative care.<br><br>*"We had a patient, [...] palliative team and everything worked, and the last dialysis was planned, but then at home he told the palliative team that he had reconsidered, he wants to continue living (laughs). [...] He is now also going back to dialysis." (Interview P01_06)*<br><br>*"Because most patients are already afraid, when we say, wouldn't it be better to have a palliative team, they associate it with everything ending quickly, so that it might come across to the patients as if it means the end, which it doesn't have to." (Interview_P01_05)*	• Patients clearly express their wishes regarding their care.<br>• Patients are cognitively able to make informed decisions about treatment continuation or end-of-life care.<br><br>*"Patients who clearly said, and who were still mentally fit at the time, who said, 'I am not coming anymore, I don't want to anymore, I am not coming anymore, I am now in a nursing home. It does not get better, it gets worse each time, and I want to die in peace.'" (Interview_P02_05)*
Relatives	• Reservations toward palliative care or cessation of life-prolonging treatments.<br>• Limited or no contact with healthcare providers.	• Involving and supporting family members to help them understand and accept palliative care or the adjustment of life-prolonging treatments.

	• Overwhelmed by dying, death, and grief; for some, these topics are taboo.<br><br>*"Relatives who are overwhelmed by death and grief and for whom it is a taboo topic." (Interview_P02_03)*	*"Integration and training of relatives: Training and integrating families so that they accept it faster, because often we come to cases where patients suffer and maybe death would be a relief, but the family out of love cannot understand, does not allow therapy to be stopped or the patient to be treated palliatively." (Interview_P03_01)*
Heath care professionals	• Limited knowledge or training in palliative care, leading to uncertainty or reluctance to address patients' palliative needs.<br>• Fear of emotional burden or difficulty handling conversations about end-of-life issues.	• Prior experience or personal comfort with communication about severe illness or palliative needs.<br>• Motivation to provide patient-centered care and respond to patients' preferences.<br>• Willingness to collaborate with colleagues and integrate palliative approaches into routine care.
